# Temporomandibular disorders in headache patients

**DOI:** 10.4317/medoral.18007

**Published:** 2012-08-28

**Authors:** Christiane-Espinola-Bandeira Mello, José-Luiz-Góes Oliveira, Alan-Chester-Feitosa Jesus, Mila-Leite-de Moraes Maia, Jonielly-Costa-Vasconcelos de Santana, Loren-Suyane-Oliveira Andrade, Jullyana-de Souza Siqueira Quintans, Lucindo-José Quintans-Junior, Paulo-César-Rodrigues Conti, Leonardo-Rigoldi Bonjardim

**Affiliations:** 1 Post graduate student – Health sciences, Federal University of Sergipe, São Cristóvão, SE, Brasil; 2 Neurologist, Federal University of Sergipe, São Cristóvão, SE, Brasil; 3 Graduate student – Medicine, Federal University of Sergipe, São Cristóvão, SE, Brasil; 4 Professor – Laboratory of Orofacial Physiology, Departament of Physiology, Federal University of Sergipe, São Cristóvão, SE, Brasil; 5 Professor, Department of Prosthodontics Bauru School of Dentistry-University of São Paulo

## Abstract

Objective: To identify the frequency of signs and symptoms of temporomandibular disorder (TMD) and its seve-rity in individuals with headache. Study Design: 60 adults divided into three groups of 20 individuals: chronic daily headache (CDH), episodic headache (EH) and a control group without headache (WH). Headache diagnosis was performed according to the criteria of International Headache Society and the signs and symptoms of TMD were achieved by using a clinical exam and an anamnestic questionnaire. The severity of TMD was defined by the temporomandibular index (TMI). Results: The TMD signs and symptoms were always more frequent in individuals with headache, especially report of pain in TMJ area (CDH, n=16; EH, n=12; WH, n=6), pain to palpation on masseter (CDH, n=19; EH, n=16; WH, n=11) which are significantly more frequent in episodic and chronic daily headache. The mean values of temporomandibular and articular index (CDH patients) and muscular index (CDH and EH patients) were statistically higher than in patients of the control group, notably the articular (CDH=0.38; EH=0.25;WH=0.19) and muscular (CDH=0.46; EH=0.51; WH=0.26) indices. Conclusions: These findings allow us to speculate that masticatory and TMJ pain are more common in headache subjects. Besides, it seems that the TMD is more severe in headache patients.

** Key words:**Temporomandibular dysfunction, headache disorders.

## Introduction

Among the most common pain in general population, stand out temporomandibular disorder (TMD) which is the most common cause of pain in the orofacial region of non-dental origin (1,2) and headache, that is a common pain symptom and an important public health problem (3-5).

The specific location of painful areas is essential for treatment planning. In addition, it is of importance whether pain is localized to joint or muscle area only or if patients frequently have pain in other areas as well (6). However, it is common associations among pain in craniocervical region. Recent studies had observed a relationship between headache and TMD (7-12) especially tension type headache and migraine (13-15). However, to the best our knowledge, there are still few studies investigating the relationship between chronic daily headache and TMD (5). Moreover, the co-occurrence of temporomandibular (TM) pain and headache may suggest a common pathogenesis, a causal association, or a common confounding factor, even though this point is yet controversial.

So, the present study intended to verify the frequency of (TM) signs and symptoms and its severity between subjects with chronic or episodic headache and those without headache at least three months ago. To the best our knowledge the present study is the first to investigate the severity of TMD, using specific tools (TMI), in headache patients.

## Material and Methods

The sample was comprised of 60 individual (both genders; 18 years old or over), 40 with headache and 20 without headache (control group) from Headache Outpatient Service in Federal University of Sergipe. Headache were diagnosed and classified by a neurologist in episodic (n=20) or chronic headache (n=20) based on the International Classification of Headache Disorders, 2nd edition (ICHD-II) (16). Control group (n=20) consisted of the asymptomatic subjects that came to the hospital with the patient (accompanying people) with no history of migraines and no headaches (even mild) over the 3 months before the study (5). Subjects gave written consent to participate in the study. The study was conducted in accordance with the current good clinical practice guidelines and it was approved by the Ethics Committee of the Federal University of Sergipe.

In order to describe individual TM symptoms, the entire sample filled out a self-reported questionnaire modified by Conti et al. (17), which contains questions about the most common TMD complaints. The results of such inventory were not used to make patient’s diagnosis, but only to compare isolated symptoms between groups.

Clinical signs of TMD was made by using some questions of a systematically translated Brazilian version of the Research Diagnostic Criteria for TMD (RDC/TMD axis I) (18), performed by an examiner previously trained and calibrated.

In addition, determination of the Temporomandibular Index (TMI) was performed (19). The TMI is composed of three sub-indices: the Functional Index (FI), the Muscle Index (MI), and the Joint Index (JI). The FI includes 12 items related to the range of motion (ROM) of the mandible, which characterize pain or limitation related to mandibular ROM and deviation of the mandible during opening movement. The MI measures pain associated with bilateral digital palpation of selected intraoral and extra-oral masticatory muscles at a total of 20 sites. The JI measures pain evoked by digital palpation of two sites for each temporomandibular Joint (TMJ) and the occurrence of noise in each TMJ.

-Statistical Analysis

GraphPad Prism version 3.0 was used for statistical analysis. Qualitative variables were presented as relative and/or absolute frequencies, while quantitative data were presented as mean values and standard deviations. The difference between qualitative variables among groups was performed using Fisher’s exact test. To compare TMI values as well as their sub-indices between headache (CDH or EH) and control group was used the Kruskal-Wallis followed by Dunn’s test. A significance level of 0.05 was set.

## Results

The results of the present study are organized according to the following criteria: characterization of sample ( Table 1), signs and symptoms of TMD in headache patients ( Table 2), frequency and severity of TMD in headache patients ( Table 3).

**Table 1:**
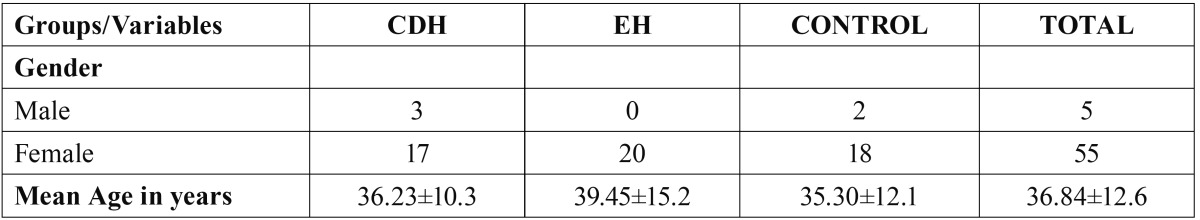
Characterization of the sample according to age and gender.

**Table 2:**
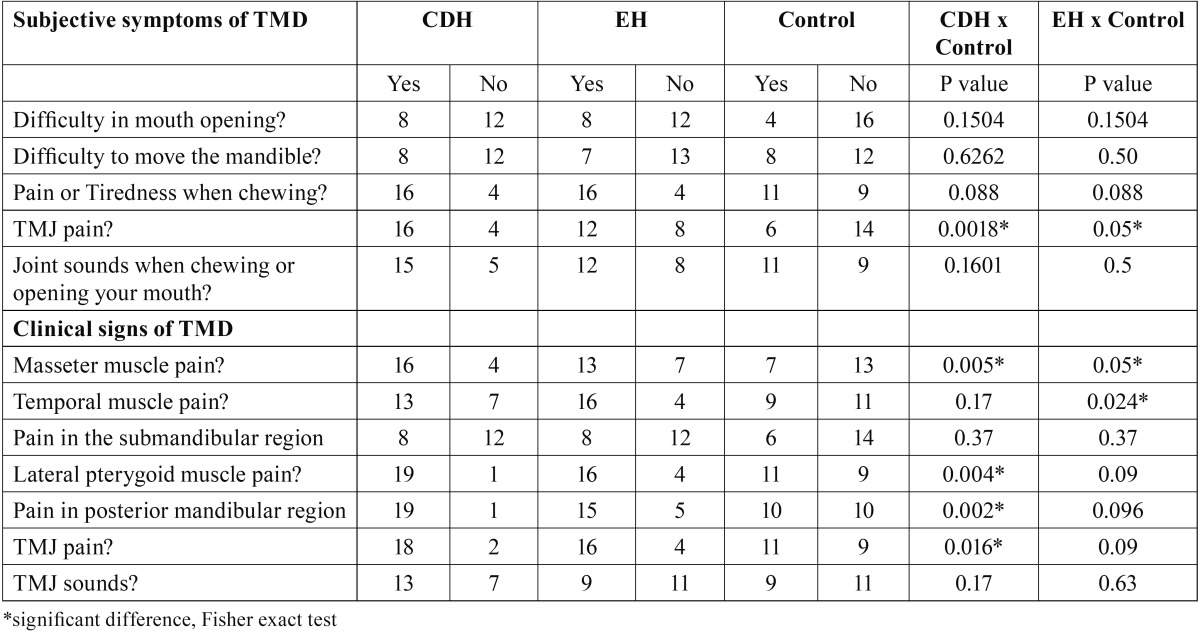
Distribution of TMD signs and symptoms between headache patients and subjects without headache.

**Table 3:**
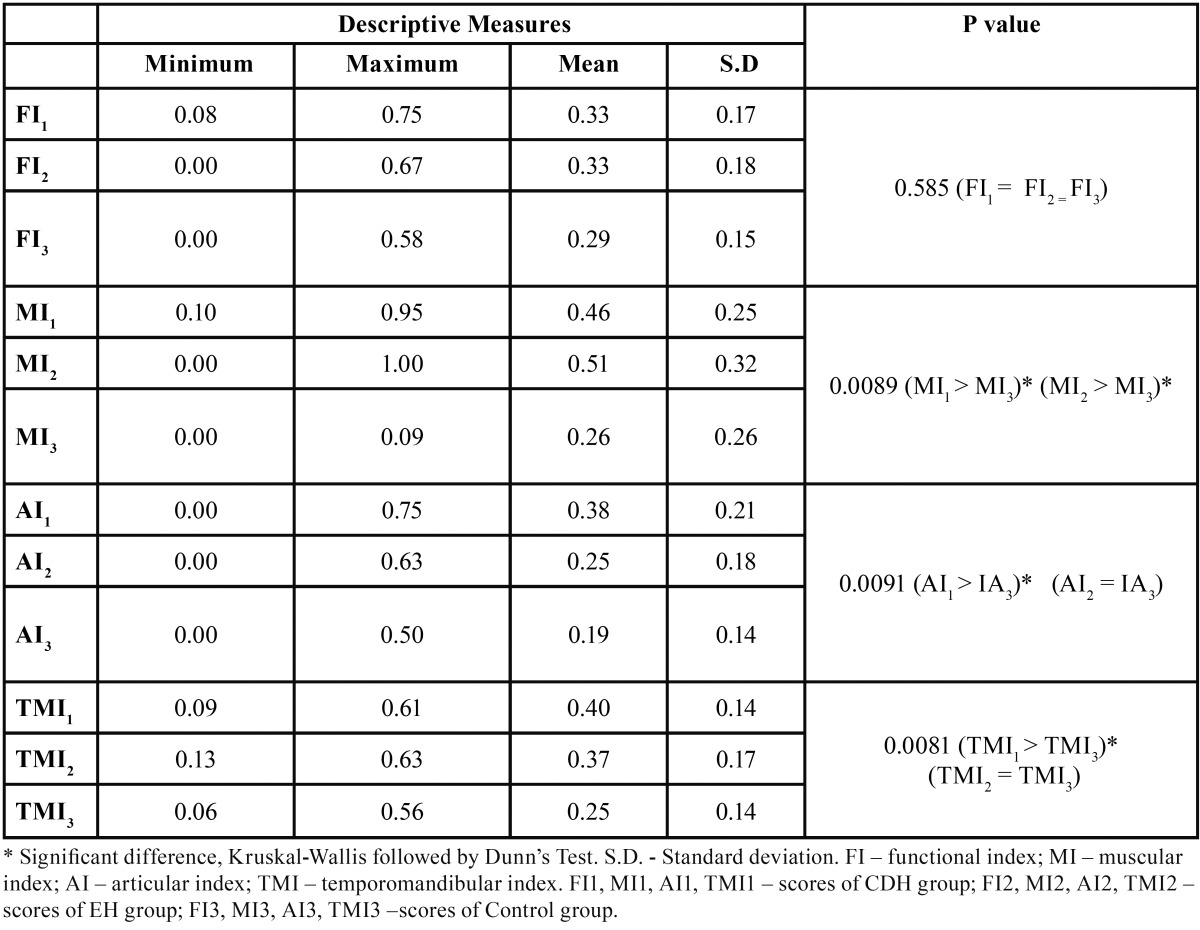
Mean values of temporomandibular index and sub-indices between headache patients and subjects without headache.

A total of 60 subjects were examined for the presence of TMD signs and symptoms. The characteristics of age and gender for all groups are showed in  Table 1. Most participants were woman (91.7%). Groups were not different regarding age and gender.

 Table 2 showed the TM signs and symptoms among the groups. It was verified that for the most of TMD symptoms, there were no statistical difference between headache groups (CDH or EH) and control group. However, TMJ pain occurred significantly more in CDH (p=0.0018) and EH (p=0.05) patients in relation to subjects without headache.

Relative to controls, subjects with CDH were significantly more likely to have clinical signs of TMD –tenderness in masticatory muscles (masseter, lateral pterigoid), pain in posterior mandibular region and TMJ pain. For EH group the clinical signs pain in masseter and temporal muscle was significantly more common related to control group (p<0.05; p=0.024).

 Table 3 shows the mean values of temporomandibular index (TMI) and subindices: Functional index (IF), Articular index (AI) and Muscular index (MI). For CDH patients the mean values of TMI, MI and AI were significantly higher when compared to control group (CDH: TMI-0.40, MI-0.46, AI-0.38); for EH patients only the mean values of MI were significantly higher than those ones of non-headache patients (MI-0.51).

## Discussion

Although the relationship between headache and TMD had already been demonstrated in several studies (5,7-12,15) the topic is still surrounded by controversy. Thus, the present study compared the frequency and severity of signs and symptoms of TMD between headache patients and subjects without headache.

To the best our knowledge, the present study investigated at the first time the severity of TM signs and symptoms according to temporomandibular index (19).

The sample of the present study was constituted basically by women (n=55), which corroborate to other studies that reported a higher prevalence of TMD (20,21) and headache (7) in women. It has been suggested that women are more sensitive than men and tend to characterize their pain as more intense, frequent, and continuous (22,23).

Related to TM symptoms, TMJ pain were significantly more frequent in headache patients (EH and CDH respectively). Additionally, the clinical signs of TMD, such as tenderness in masticatory muscles and TMJ pain were also statically more common in headache patients, especially those ones with CDH in relation to subjects without headache, corroborating with others studies that also found a higher prevalence of masticatory muscle tenderness and TMJ pain in headache patients (5,7,10,15,24,25).

It was verified scores significantly higher for TMI and their subgroups MI and AI among CDH patients and for MI in EH subjects. These findings suggest that TMD severity, especially those related to TMJ and masticatory muscle pain, are higher in headache patients, mainly with chronic condition. This allows us to suggest that, even though TMD is common among the general population, its severity is higher in patients with headache. There is no consensus in the literature regarding the cause and effect relationship between TMD and headache, given that some studies report that chronic and episodic headache patients are more prone to TMJ and masticatory muscles pain (5), thus suggesting these headaches are a risk factor for TMD, and other studies imply the contrary, that TMD may stimulate the appearance of headaches (10).

It is well established that neurons in the trigeminal system (e.g. trigeminal nucleus caudalis) integrate nociceptive input from TMJ and masticatory muscles as well as cranial tissues. Graff-Radford (26) contends that TMD elicit or exacerbate headache because of an overlap of innervations with the trigeminal nerve.

Thus, it is possible that headache facilitated (peripheral and central sensitization) masticatory muscle and/or TMJ pain or these pain conditions could be a trigger of headache which is also suggested by Stuginsky-Barbosa et al. (5) and Gonçalves et al. (15). Data of the present study could indicate that the pain reported by TMD and headache patients share a similar etiologic mechanism (9).

Despite the fact that knowing the temporal relationship between headache and TMD is difficult, in the present study the patients were informally asked about this relationship. The most of them related that the headache appeared before pain from TMD, so it is tempting to speculate headache as triggering factor to TMD development. Accordingly, pain from masticatory muscles and TMJ may act as a perpetuating and/or aggravating factor in headache patients. Bevilaqua-Grossi et al. (11) cited that TMD could be associated to transformation of episodic headache in chronic headache. In this sense, a study indicated that TMD treatment may be beneficial for individuals with headache (8).

These findings allow us to suggest that masticatory and TMJ pain are more common in headache subjects. Besides, it seems that the TMDs are more severe in headache patients. Therefore, in cases in which the neurological treatment fails to eliminate the headache, it is suggested a thorough evaluation of the stomatognathic system, given that headache patients can benefit from evaluation and treatment of TMD.

Finally, the data indicates the need for a multidisciplinary diagnosis and treatment of headache patients, given that the associated treatment of headache and TMD could bring more benefits regarding the symptoms relief of such individuals.
